# Advances in γδ T Cells: Cutaneous Biology and Therapeutic Potential for Skin Neoplasms

**DOI:** 10.1111/1346-8138.70461

**Published:** 2026-08-21

**Authors:** Simo Du, Anika Jallorina, Toru Yoshino, Lingyu Bao, Han Bao, Victor Chang, Henry K. Wong, Derek V. Chan, Stephen K. Richardson, Mary F. Lee‐Wong

**Affiliations:** ^1^ Department of Medicine Jacobi Medical Center, Albert Einstein College of Medicine Bronx New York USA; ^2^ Department of Medical Oncology Montefiore Medical Center, Albert Einstein College of Medicine, Montefiore Einstein Comprehensive Cancer Center Bronx New York USA; ^3^ California University of Science and Medicine Colton California USA; ^4^ Department of Medicine, Hematology/Oncology Rutgers New Jersey School of Medicine Newark New Jersey USA; ^5^ Section of Hematology/Oncology Veterans Administration New Jersey Health Care System East Orange New Jersey USA; ^6^ Department of Dermatology UC San Diego School of Medicine San Diego California USA; ^7^ All Dermis Dermatology New York New York USA; ^8^ Department of Dermatology Icahn School of Medicine at Mount Sinai New York New York USA; ^9^ College of Medicine, University of Central Florida Orlando Florida USA; ^10^ University of Central Florida HCA Florida Healthcare Graduate Medical Education Tallahassee Florida USA; ^11^ Icahn School of Medicine at Mount Sinai New York New York USA

**Keywords:** cellular therapy, gamma delta T cell, melanoma, skin cancer, tumor microenvironment

## Abstract

γδ T cells represent an increasingly important subset of T lymphocytes in the immune response to cancer and as a platform for novel immunotherapies. The aim of this review is to synthesize recent insights into the biology and advances in therapeutic applications of γδ T cells in cutaneous malignancies, especially melanoma, within a framework of tumor immunology and recent clinical research.

## 1 Introduction

The existence of γδ T cells was inferred from the discovery of γδ genes [1]. During the double‐negative stage of T cell development, the rearrangement of γ, δ, and β TCR chains occurs in parallel, followed by α chain rearrangement (during the double‐positive stage). Lineage commitment to either the αβ or γδ T cell phenotype depends on factors that influence the timing of rearrangement events and gene locus. In humans, γδ T cells are predominantly double‐negative cells as the majority do not express either CD4 or CD8 coreceptors. γδ T cells are included in the family of unconventional T cells [2]. (Figure 1).

**FIGURE 1:**
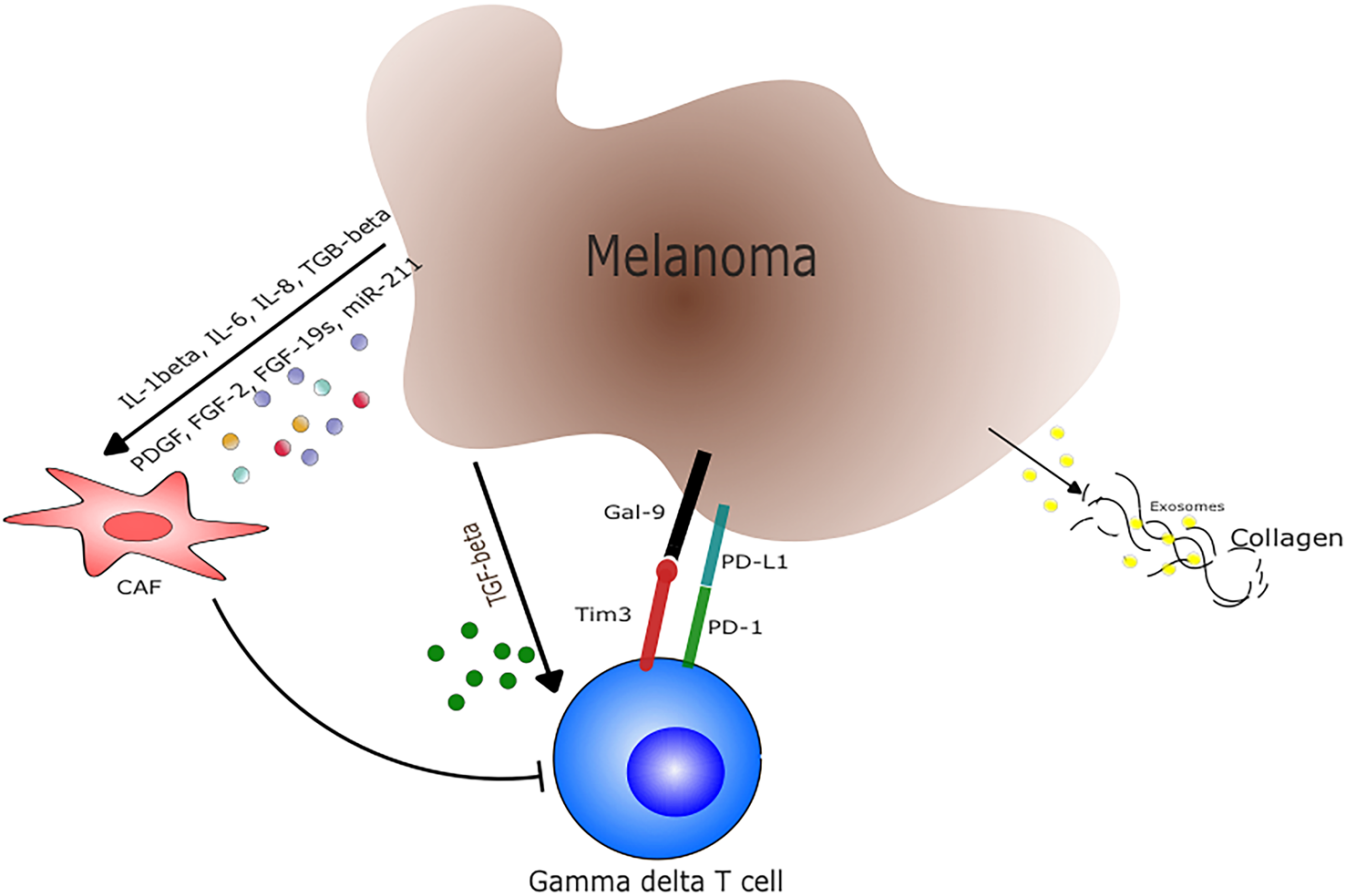
Melanoma tumor microenvironment and the effects on γδ T cells. FGF, fibroblast growth factor; miR, microRNA; PDGF, platelet‐derived growth factor.

The human TCRγ locus consists of 12 functional Vγ genes, and the TCRδ locus consists of 8 Vδ genes, allowing for the potential assembly of over 10 [3] unique γδ T cell receptors. The diversity of the TCR repertoire exhibited by γδ T cells varies based on their tissue localization; epithelial γδ T cells exhibit a less diverse TCR repertoire than γδ T cells in the blood/lymphatics [4]. Additionally, Vδ2 cells account for the majority of γδ T cells in the peripheral blood, whereas Vδ1 cells account for the majority in epithelia.

These tissue‐specific populations also exhibit differences in their cytokine profiles. Differences between epidermal and non‐epidermal (peripheral) γδ T cells have been studied. Epidermal γδ T cells produce greater levels of IL‐4, −8, −13, MIP‐1β than the peripheral subset, whereas both populations produce similar levels of GM‐CSF, IFN‐γ, MIF, MIP‐1α, and CCL5. This suggests that epidermal γδ T cells may be more likely to exhibit a Th2‐type response than peripheral blood γδ T cells [5]. Irrespective of their tissue localization, these are all also subsets of γδ T cells that express NKG2A and FcRγIII (CD16) receptors, with implications for their recognition and response to tumor antigens and antibodies.

## 2 Methods

From July 2025 to November 2025, a structured literature search was conducted in PubMed to identify relevant studies on γδ T cells in melanoma and cutaneous oncology. The following search was conducted: (gamma delta t cell) AND (melanoma) OR (squamous cell carcinoma) OR (basal cell carcinoma) OR (Merkel cell carcinoma) OR (primary cutaneous lymphoma). This resulted in a total return of 44 873 results. All article types were included. Only articles in English were included. Article selection was screened for relevance to γδ T cell biology, prognostic significance, or therapeutic applications in the previously listed malignancies. Articles were excluded if they did not discuss γδ T cells or their roles or applications in the previously mentioned malignancies. A total of 76 articles were included in the final analysis.
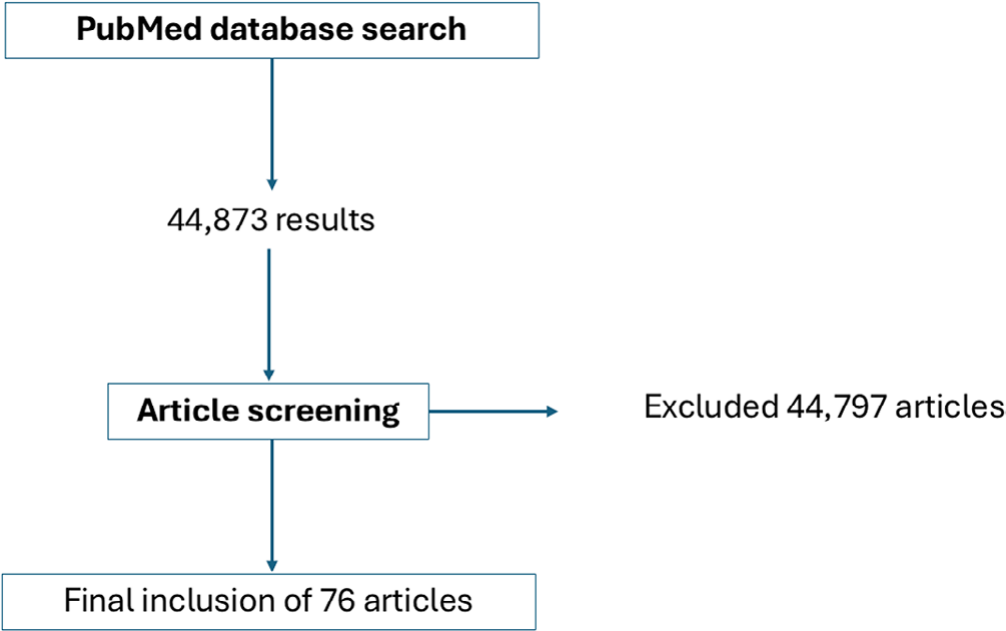


## 3 Results

### 3.1 γδ T Cell Subsets

Aside from tissue‐specific subsets, γδ T cells can also be categorized based on their Vγ/Vδ gene expression profile, T cell repertoire, cytokine profile, and/or maturation status. Single‐cell sequencing techniques identified 12 distinct subsets, with Vδ1 cells displaying tissue‐resident regulatory properties and Vδ2 cells showing cytotoxic specialization [6]. Functional subsets include IFN‐γ and IL‐17‐producing variants.

Vδ1 cells reside in the epithelium and offer a more robust immune system response compared to Vδ2 cells. They have a large TCR repertoire that is broad at birth and becomes more specific over time. As they mature, Vδ1 cells lose their CD27 expression while retaining their antigen sensitivity, cytolytic activity, and tissue specificity. The observed persistence of these cells in cutaneous and mucosal sites suggests an important role in adaptive immune responses [7].

Vδ1 cell expansion involves both chemokine pathways and antigen‐mediated cues to target carcinogenic tumors [8]. Their trafficking into tumor microenvironments is mediated by inflammatory chemokine pathways, such as the CCR2/CCL2 axis. In this axis, γδ T cells adapt into type 1 cytotoxic γδ cells that release IFN‐γ to produce an anti‐tumor effect. This coincides with the more adaptive immune surveillance of clonal selection. Malignancies may be targeted through Vδ1 cell expansion, which may occur through both chemokine‐ or antigen‐mediated pathways [8].

Vγ9δ2 cells recognize phosphoantigens and are associated with cancer surveillance and elimination [1]. Vγ9δ2 TCR activation is initiated primarily through phosphoantigen recognition. Phosphoantigens are pyrophosphorylated metabolites of isoprenoid synthesis derived from host or microbial origin. These metabolites may be elevated following aminobisphosphonate administration and decreased with HMG‐Co‐A reductase inhibitors [9]. When the phosphoantigen binds to the intracellular BTN3A1‐B30.2 domain in the Vγ9δ2 T cell, the B30.2 domains and BTN2A1 form a complex. The formation of this complex leads to BTN2A1‐IgV and Vγ9‐TCR binding, resulting in T‐cell activation [10]. The BTN2A and BTN3A genes are widely expressed and are induced by IFN‐γ.

Unlike αβ T cells, γδ T cells are MHC‐independent and able to recognize non‐polypeptide antigens such as MR1, CD1a, and isopentenyl pyrophosphate. The 2 peptide chains of the γδ T cell receptor are associated with 3 CD3 dimeric subunits: CD3εγ, CD3εδ, and CD3ζζ. The TCR peptide chains and the CD3γ, CD3δ, and CD3ε each contain an extracellular domain. Binding of the peptides leads to phosphorylation of a CD3‐associated intracellular immunoreceptor tyrosine kinase activation motif (ITAM) and downstream effector function. Two groups recently reported the cryo‐EM structure of two γδ TCR clones, G115 (Vγ9δ2) and 9C2 (Vγ5δ1) [11, 12]. Compared to the αβ TCR, the extracellular domains of the γδ chains do not have stabilizing interactions with the CD3 chain and are much more mobile, perhaps explaining some of the increased range of antigen recognition [13].

In addition, MHC class I (MHC‐I) polypeptide related sequence A (MICA) and MHC‐I polypeptide related sequence B (MICB) expression, normally low in healthy states, are upregulated during cellular stress and during conversion to tumor cells [14]. MICA and MICB may activate both natural killer (NK) cells and γδ T cells, specifically the VγV9δ2 subset, via NKG2D on γδ T cells, resulting in cytokine release [14]. ULBP4 is another high‐affinity ligand that contributes to the immune surveillance effect of γδ T cells. ULBP4 expressed on cancer cells has the ability to bind TCRγ9/δ2, resulting in γδ T cell activation and consequent Th1 cytokine secretion, playing a role in the anti‐tumor effects [15]. hMSH2 works similarly to ULBP4 in that its binding to Vδ2 TCRs leads to γδ T cell activation, playing a role in tumor immune surveillance [16].

While many of the ligands are unknown, the interaction of phosphoantigens and butyrophilins has been studied intensively. Phosphoantigens are produced by various microorganisms and are also intermediates in the farnesyl diphosphate synthase pathway (FDSP). Changes in FDSP activity can lead to activation by accumulation of phosphoantigens. Bisphosphonates can interfere with this pathway and increase isopentenyl phosphate levels and activate γδ T cells. Conversely, statins can decrease levels through HMG‐CoA reductase and decrease γδ T cell activation [9]. As previously mentioned, phosphoantigens interact with the BTN2A1‐B30.1 dimer and BTN3A1 B30.2 domain to bind and activate the γδ T cell receptor [17].

γδ T cells exert direct cytotoxicity by releasing cytolytic granules such as perforin and granzymes and induce apoptosis through TRAIL and FasL pathways [18]. γδ T cells express both Perforin and Perforin‐2, which are proteins of the innate immune system that function to destroy target cells via pore formation [19]. In addition, they secrete IFN‐γ, TNF‐α, and chemokines that crosstalk with NK cells, dendritic cells, and αβ T cells, thereby amplifying broader anti‐tumor immunity.

γδ T cells interact with other immune cells, including αβ CD4+ cells, αβ CD8+ cells, neutrophils, macrophages, NK cells, and B cells. In co‐culture experiments, plasmacytoid dendritic cells and γδ T cells were able to stimulate each other's maturation in a process requiring soluble factors and membrane contacts. Also, dendritic cells may be recruited by γδ T cells by the release of Granulocyte‐Macrophage Colony‐Stimulating Factor (GM‐CSF). IFN‐γ released by γδ T cells results in MHC‐I and CD86 expression on dendritic cells, promoting T‐cell activation [20].

T cell exhaustion, a state of dysfunction that arises from chronic antigen exposure and persistent stimulation within the tumor microenvironment (TME), plays an important role in tumor progression. Exhausted γδ T cells express a suite of these immune‐inhibiting receptors, including Programmed Cell Death Protein 1 (PD‐1), Lymphocyte‐Activation Gene 3 (LAG‐3), and T cell Immunoglobulin and Mucin‐domain containing‐3 (TIM‐3), along with diminished cytokine production (e.g., IFN‐γ) and impaired cytotoxic capacity [3].

A distinct subset, γδ T17 cells, secrete IL‐17, which can drive tumor progression through multiple mechanisms including angiogenesis, recruitment of immunosuppressive neutrophils, and activation of tumor‐associated macrophages. These effects have been demonstrated in preclinical murine models [21]. Hu et al. mention that the protumor effect of these IL‐17‐secreting γδ T cells in human and mouse models has contributed to our current knowledge of their role in tumorigenesis [22].

### 3.2 Tumor Infiltrating Lymphocytes in Cancer

Tumor infiltrating lymphocytes (TILs) usually include natural killer cells, macrophages, neutrophils, dendritic cells, helper CD4+ T cells, cytotoxic CD8+ cells, regulatory T cells, and B cells. The study of TILs has led to a new FDA‐approved therapy for metastatic melanoma [23, 24]. However, even though the bulk of antitumor cytotoxic activity in most solid tumors involves conventional CD8+ T cells, research in TILs has not always included analysis of γδ T cells, and their role as TILs remains unclear.

In primary melanoma, a higher number of TILs in melanoma is associated with improved prognosis. In a series of 4 957 patients from 1993 to 2024, known TIL and outcomes data were analyzed. TILs were prognostic of melanoma‐specific, overall, and recurrence‐free survival [25].

In a large study of gene expression in 39 malignancies and 22 leukocyte subsets of infiltrating immune cells of more than 18 000 patients, γδ T cells had the most favorable prognostic association. Plasma cell signatures were of significance as well, being associated with favorable survival for solid tumors [26]. As a study of bulk tissue, it was unable to distinguish cells from tumor vs. cells from TME. In one study that compared TILs from patients with pancreatic, ovarian, bladder cancer, and melanoma, gene expression of butyrophilin 3A (BTN3A) cells, a marker of γδ T cells, was increased for melanoma and bladder cancer and associated with improved outcome [27].

Another study that compared TILs across a variety of cancers from The Cancer Genome Atlas (TCGA) reported improved overall survival among patients who exhibited greater diversity in their TCR repertoire. Additionally, a direct correlation was observed between the degree of TCR clonality and tumor responses to anti‐PD‐1 immunotherapy [28]. Among TILs in 471 melanoma patients in the TCGA, 1 121 γ clones were identified; γ and δ TCR gene expression was associated with improved survival among these patients [29].

While γδ T cells typically play a cytotoxic role in antitumor immunity, there is also a small subset of γδ T cells that exhibits regulatory properties. Because the TME contains immunosuppressive cytokines (e.g., TGF‐β1, IL‐10), it has been hypothesized that these cytokines promote the development of regulatory γδ T cells. Studies revealing the regulatory function of γδ T cells in breast and colorectal cancer support the notion of functional plasticity in this population [30]. It is worth noting that Vδ1+ γδ T cells, which are more concentrated in epithelial tissues, exhibit substantial FoxP3 expression when stimulated in vitro, as opposed to Vδ2+ T cells under similar conditions [31]. This suggests that Vδ1+ rich environments, such as the epithelium, have an enriched capacity for γδ T cell regulatory function. See Table 1 for a summary of functions of γδ T cells by skin cancer type.

**TABLE 1:** Γδ T‐cell functions by different skin cancer types.

Feature	Malignant melanoma	Squamous cell carcinoma (SCC)	Basal cell carcinoma (BCC)	Merkel cell carcinoma
**Typical Infiltration Level**	Present, can be abundant	Enriched compared to normal skin	**Depleted** compared to normal skin	Variable, present in ~50% of tumors
**Dominant Subsets in TME**	Vδ1+ and Vδ2+	Vδ1+ and Vδ2+	Undetermined due to low frequency	Predominantly Vδ2^+^ subset
**Early stage**	Vδ2^+^ cells predominate	IFN‐γ‐producing Vδ1^+^/Vδ2^+^ cells enriched	Minimal data; γδ cells rare and function poorly defined	High γδ infiltration correlates with better outcome
**Late stage**	Vδ2^+^ frequency drops; skewed toward regulatory/Th17 profiles	Shift to IL‐17‐producing γδ cells (γδ17 phenotype)	Persistently depleted	Vδ2^+^ cells show PD‐1^+^ LAG‐3^+^ exhaustion
**Primary Functional State**	Functionally impaired/exhausted	**Stage‐dependent:** IFN‐γ producing (early) vs. IL‐17 producing (advanced)	Likely suppressed or excluded	Mixed exhausted yet immunoreactive state
**Key Cytokine Signature**	Low IFN‐γ/TNF‐α; potential for IL‐17	IFN‐γ (early) vs. IL‐17 (advanced)	Low overall; TME is TGF‐β rich	IFN‐γ and TNF‐α when revived by PD‐1 blockade
**Prognostic Significance**	Vδ2+ infiltration linked to better outcome; Vδ1+ predicts ICI response	IFN‐γ producing cells (favorable); IL‐17 producing cells (unfavorable)	Poorly defined; depletion suggests a lack of protective immunity	High γδ infiltration associated with better survival and ICI response; potential prognostic biomarker
**Role in ICI Therapy**	Direct targets; express PD‐1, LAG‐3, TIM‐3; Vδ1+ presence predicts response	Potential targets; not as well‐defined as in melanoma	Unlikely to be primary targets due to low abundance	Strong predictor of PD‐1/PD‐L1 therapy benefit; γδ infiltration correlates with tumor control

### 3.3 γδ T Cells and the Tumor Microenvironment (TME)

Much can be gleaned from the role of γδ T cells in the TME using melanoma as a model. (Figure 2) Research in this area has enhanced our understanding of the paradoxical role of γδ T cell subsets in both tumor progression and resolution. The functional properties of these cells are heavily influenced by the cytokine milieu in addition to their interactions with other lymphocytes, antigen‐presenting cells, fibroblasts, and myeloid‐derived suppressor cells (MDSCs) [32]. Figure 3 depicts some key differences of γδ T cells in the normal microenvironment vs. the TME.

**FIGURE 2:**
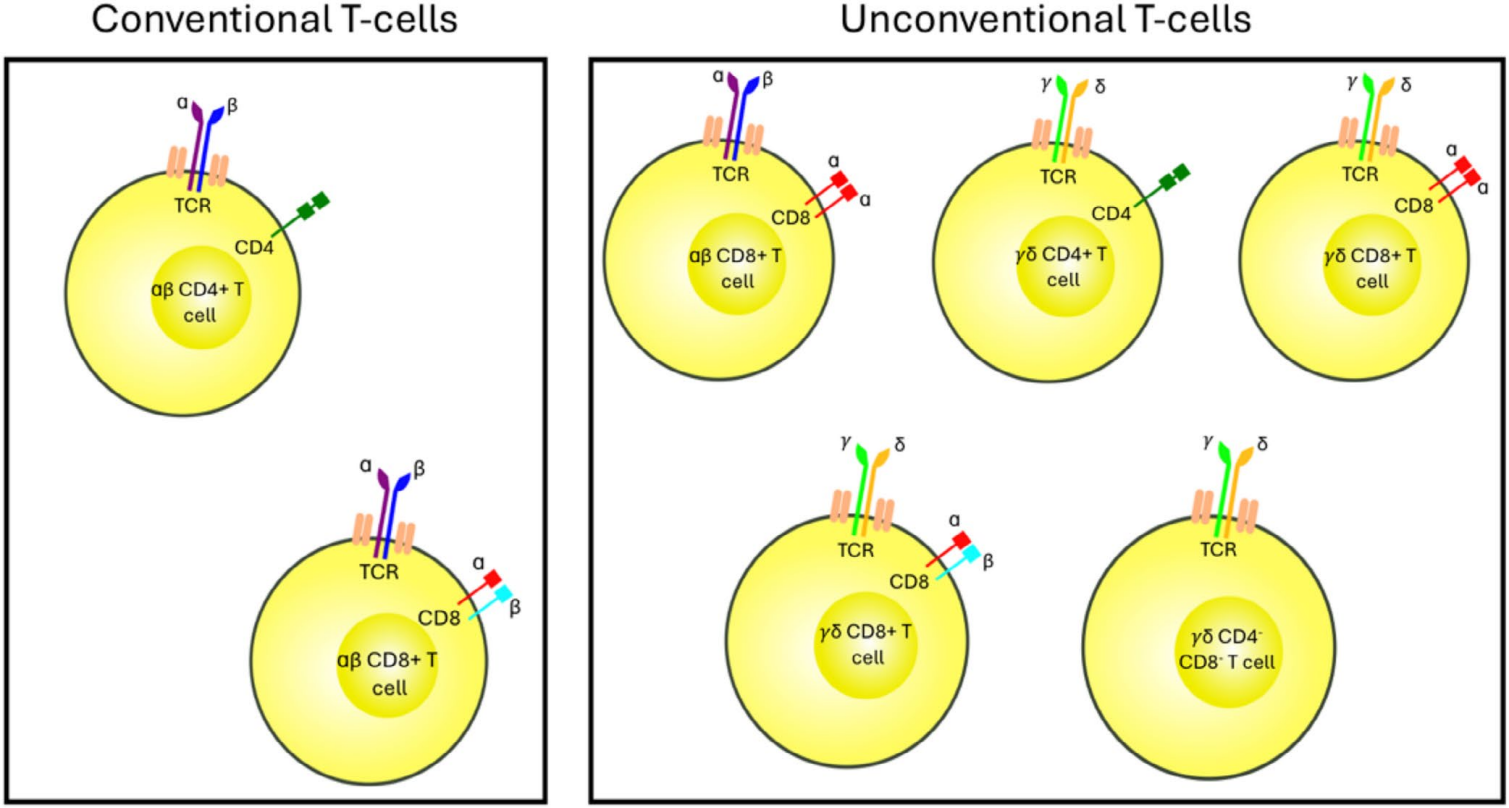
Conventional and unconventional T‐cell subset variations by TCR, CD4, and CD8 receptors. TCR, T‐cell receptor.

**FIGURE 3:**
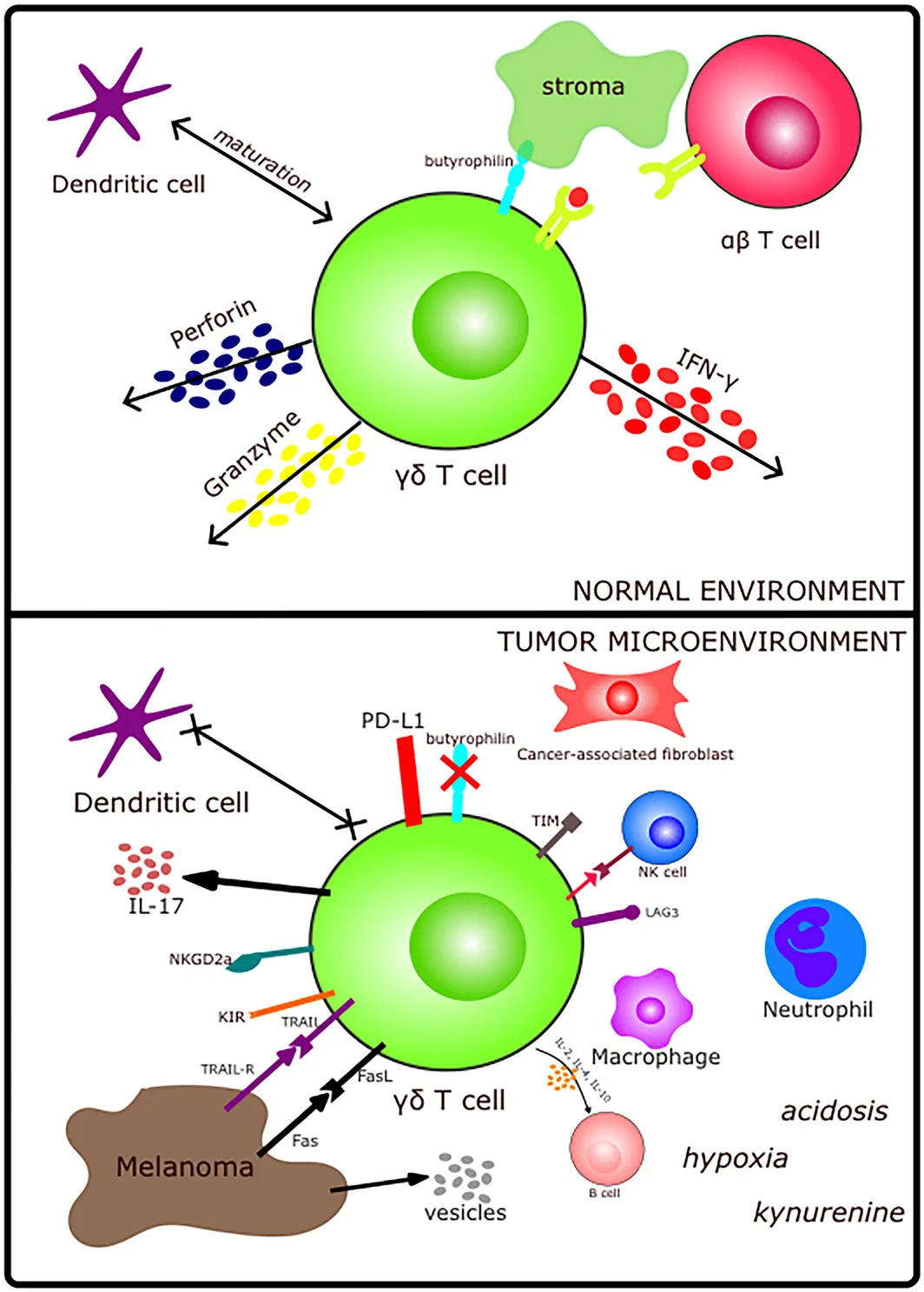
γδ T‐cells in normal environment vs. tumor microenvironment.

Tumor cells along with lymphocytes and cancer‐associated fibroblasts (CAFs) contribute to the cytokine‐rich immunosuppressive microenvironment of melanoma. In particular, TGF‐β and IL‐10 promote the differentiation of γδ regulatory T cells, which in turn produce FoxP3, IL‐10, TGF‐β, and ectonucleotidases (including CD39 and CD73). The suppression of βF‐1+ T cell and dendritic cell activity by these cytokines promotes the development of MDSCs that further dampen anti‐tumor immunity. Regulatory γδ T cells are also believed to play a similar role in the setting of breast, colorectal, and pancreatic cancer, among others [33].

Interestingly, different γδ T cell subsets have been reported to exhibit paradoxical effects in the melanoma TME in vitro. Vδ1+ cells in humans and Vγ4+ cells in mice in particular have been shown to exhibit antitumor properties. Li et al. noted that Vδ1+ T cells kill melanoma cells via the perforin‐granzyme B, TRAIL, and Fas/FasL pathways [18]. Vγ1+ cells, however, were reported to have a suppressive effect on Vγ4+ cells [34].

While we discuss the prognostic significance of TILs in various skin neoplasms, it is important to note that it is not only the quantity of TILs that may be indicative of prognosis, but also their functional state, subsets present, and differentiation profile. Studies are emerging that illustrate the varying functional states of γδ T cells, such as regulatory, cytotoxic, and exhausted phenotypes. Each of these functional states impacts clinical outcomes and tumor suppression or progression. Thus, sole quantitative characterization of γδ T cells is not sufficient to determine their biological impact.

### 3.4 Melanoma

The high tumor mutational burden in melanoma has led to various research efforts in immunotherapy for this disease. Recent studies have established that a higher number of TILs in melanoma is a factor associated with improved prognosis [35]. In particular, a study demonstrated a relatively increased proportion of γδ T cells in the TIL population [36]. These cells are now believed to play an important role in antitumor immunity and progression‐free survival in this disease setting.

Cordova et al. investigated surgically removed primary melanoma and found that γδ T cells represented 50% of TILs. Among these cells, 37% were CD4 + CD8‐, and 13% were CD4‐CD8 + [35]. In vitro studies, the production of IFN‐γ and TNF‐α by polyclonal γδ T cells derived from the primary melanoma resulted in the killing of melanoma cell lines, thus supporting their role in antitumor immunity. The majority of these γδ T cells that infiltrated these tumors demonstrated an effector memory and terminally differentiated phenotype [35].

Regarding disease staging, a higher percentage of γδ T cells has been reported among TILs in patients with early melanoma (Stages 0–II) compared to those with more advanced disease (Stages III–IV) [35]. While the majority of γδ T cells infiltrating melanoma consists of Vδ1+ cells, Vδ2+ T cells, which are largely absent in normal skin [37], were found in increased numbers, suggesting enhanced trafficking from peripheral blood and/or lymphoid tissues to the TME [35]. A study by Toia et al. observed a notable reduction in circulating Vγ9δ2 T cells among patients with advanced disease, suggesting a role for this subpopulation in the immunologic response to melanoma [38]. The percentage of Vγ9δ2 T cells present among TILs also correlated with mortality and disease relapse. More specifically, a reduction in disease relapse (13.0% when present, 35.7% when absent, *p* = 0.045) and mortality (0% when present, 21.4% when absent, *p* = 0.005) were noted at a median follow‐up of 36 months [38]. Other studies have reported improved progression‐free survival among melanoma patients with higher levels of circulating Vδ2+ cells, suggesting this population may serve as a potential prognostic biomarker in the near future [39].

Lastly, ICI resistance has been associated with interferon signaling and defects in presenting antigens. Such defects include loss of β2‐microglobulin, downregulation of HLA‐I, and JAK/STAT pathway defects, which lead to lower responsiveness to IFN‐γ. Such adjustments in immune system function limit αβ T‐cell recognition and may contribute to pro‐tumor properties.

### 3.5 Squamous Cell Carcinoma (SCC)

γδ T cells represent a small proportion (< 5%) of TILs associated with cutaneous squamous cell carcinoma (cSCC); however, they notably exhibit functional plasticity as the disease progresses from early to more advanced stages [40]. Among 47 cSCC patients reported by Lo Presti et al., patients with early‐stage disease exhibited a larger percentage of IFN‐γ‐producing γδ T cells, which was associated with lower mortality rates, a reduced risk of relapse, and decreased incidence of nodal metastases. Conversely, those with more advanced disease (Stage III‐IV) exhibited a larger proportion of IL‐17‐producing Vδ1 and Vδ2 and increased mortality [40]. Additionally, researchers found that Tregs were more abundant in TME than the peripheral blood. This finding correlated with more advanced disease, suggesting that the proportion of Tregs and γδ T cells may serve as prognostic indicators for cSCC [40].

Recent work by Alexander et al. proposed a mechanism through which γδ T cells promote the destruction of tumor cells and perhaps may serve as an avenue for the development of γδ T cell‐based immunotherapeutics [41]. More specifically, they induced the expression of CD56 on γδ T cells in vitro. These cells were capable of killing SCC cell lines through cytotoxic degranulation, consistent with the elevated CD107a levels they detected among this population [41].

### 3.6 Basal Cell Carcinoma (BCC)

Limited research exists on the role of γδ T cells in BCC, as they are nearly absent in the TME of this low‐grade malignancy. More specifically, γδ T cells account for only about 1% of lymphocytes isolated from these tumors, even lower than the 2% reported in normal skin (*p* = 0.002) [42]. This suggests that the TME may not be conducive to the retention, recruitment, and/or survival of these cells in this disease setting. This pattern of immune exclusion is fundamentally different from the infiltration and subsequent reprogramming seen in melanoma and SCC, perhaps due to the fact that BCC infiltration is more superficial than that of SCC and melanoma. Peculiarities unique to the BCC TME remain to be elucidated.

### 3.7 Merkel Cell Carcinoma (MCC)

MCC is an aggressive malignancy characterized by immune dysfunction. This notion is supported by its high clinical response to anti‐PD‐1/PD‐L1 immunotherapy. Both pembrolizumab and avelumab have shown efficacy in the treatment of MCC by reversing the immune dampening effects of PD‐1 expressed by exhausted T cells in the TME [43, 44]. It has also been observed to occur with higher likelihood in an immunosuppressive state, but spontaneous tumor regression is thought to be due to immune clearance [45].

The role of γδ T cells in MCC has yet to be formally characterized; however, their contribution to the lymphocytic tumor infiltrate is notable. Gherardin et al. analyzed the T‐cell infiltrates of MCC lesions in 39 patients. Almost 50% of these tumors exhibited a prominent CD3 + CD4‐CD8‐ double negative T‐cell population, representing the largest percentage of the lymphocytic infiltrate in ~12% of cases. Among this group, the majority were Vδ2+ cells that also expressed PD‐1 and LAG3, well‐established indicators of T‐cell exhaustion [46] and a target for immunotherapy. Although these findings suggest that γδ T cells are involved in the response of MCC to immune checkpoint inhibitors (ICIs), the current evidence must be reinforced with further studies. Currently, CD8+ αβ T cells play the primary role in MCC response to ICIs. Further, γδ T cells may play a complementary role to these CD8+ αβ T cells rather than acting as the primary mediator of ICI therapy response.

### 3.8 A Note on B Cell Lymphoma

The first reported case of primary cutaneous malignant B cell lymphoma with abundant γδ T cells was published in 2014, with prevalent γδ T cells located within the primary skin lesion and peripheral blood [47]. γδ T cells have good utility against B cell lymphomas, being able to have a potent cytotoxic effect against tumors that downregulate MHC, as they are independent of MHC antigen presentation. They also act regardless of mutational burden, allowing anti‐tumor effects against tumors with even just a few somatic mutations [48].

### 3.9 Primary Cutaneous γδ T‐Cell Lymphoma (PCGD‐TCL)

Primary cutaneous γδ T‐cell lymphoma (PCGDTCL) is an uncommon malignancy characterized by the abnormal proliferation of γδ T cells in the skin without evidence of extracutaneous disease at the time of initial diagnosis. It usually presents as ulcerated thick plaques and tumor nodules favoring the lower extremities. In most cases, the disease is rapidly progressive with a 10% 5‐year overall survival [49].

Histologically, it may present with predominantly epidermal, dermal, or subcuticular involvement [50]; however, it is not unusual for more than one pattern to be observed in the same tumor specimen. Additionally, there is often notable angiocentricity with associated invasion and destruction of blood vessels.

The majority of cases exhibit a CD3 + CD56 + CD4‐CD8‐ phenotype with Vδ1+ cells accounting for the majority of the malignant infiltrate in cases limited to the skin. Cases involving the subcutis primarily consist of Vδ2+ cells and are associated with a worse prognosis [51], particularly in the setting of hemophagocytic syndrome. As these subtypes normally compartmentalize to these locations, the predominance of Vδ1+ cells in skin and Vδ2+ in the subcutis is not surprising.

Of note, other CTCLs, such as mycosis fungoides, lymphomatoid papulosis, and primary cutaneous anaplastic large cell lymphoma (pcALCL), may uncommonly be associated with γδ TCR expression, but such cases generally do not differ from βF‐1 positive variants in their clinical course or prognosis [52].

### 3.10 γδ T Cells and Immunotherapy: Historical Background and Evolving Therapies

While γδ T cells migrate into tumor tissue to fight the tumor, these infiltrating γδ T cells typically show or develop an exhausted phenotype in the TME, with an increased number of immune checkpoint markers on the cell surface. Targeting the immune checkpoint receptors on the surface of these cells may prove to be an effective strategy for reversing T‐cell exhaustion and augmenting the antitumor response through better γδ T cell activation, proliferation, and cytotoxic ability [53].

Melanoma has long served as an established prototypic cancer for immunotherapy. Decades before checkpoint inhibitors and adoptive TIL therapy reshaped clinical practice, clinicians explored immune‐based strategies aimed at both innate and adaptive immunity. One of the earliest examples was the use of intralesional *Bacillus Calmette–Guérin* (BCG) for patients with in‐transit melanoma in the 1970s. Although initially viewed as a nonspecific immune stimulant, later mechanistic work demonstrated that BCG produces phosphoantigens capable of activating circulating Vγ9δ2 γδ T cells, the predominant subset in humans. In regressing melanoma lesions, infiltration by Vγ9δ2 cells was identified, providing some of the earliest clinical evidence that γδ T cells could be engaged therapeutically in this disease [54].

These observations brought attention to the distinctive biology of γδ T cells. In contrast to conventional αβ T cells, which rely on MHC‐restricted peptide recognition, γδ T cells detect stress‐induced ligands and non‐peptide antigens in an MHC‐independent manner [55]. Because γδ T cell recognition occurs independently of MHC, these T cells can maintain an antitumor effect even when melanoma cells escape αβ T‐cell recognition. Despite this, current literature showing effective melanoma therapeutics with MHC‐I loss remains scarce [56]. Early translational trials attempted to exploit this biology pharmacologically; aminobisphosphonates, such as zoledronate, given with low‐dose IL‐2, reproducibly expanded Vγ9δ2 cells in patients with advanced melanoma and drove their tumor infiltration. While these interventions confirmed γδ activation in vivo, objective tumor regressions were infrequent, highlighting the limitations of single‐agent approaches (Table 2) [57, 58].

**TABLE 2:** Clinical trials & translational experiments of γδ t cells in melanoma by chronological order.

Year/Era	Intervention	Mechanism of γδ T‐cell Engagement	Patient Population/Phrases	Key Findings & Outcomes	Trial Identifier/References
1970s–1980s	Intralesional Bacillus Calmette–Guérin (BCG)	BCG induces phosphoantigens → strong activation of Vγ9δ2 T cells	In‐transit or cutaneous melanoma	Regression of injected lesions; histology showed γδ infiltration; early proof of γδ responsiveness	Yang et al., 2017 (PMID: 28151412)
1990s–2000s	In vitro expansion of γδ T cells (ex vivo IL‐2 ± phosphoantigens)	Autologous expansion and adoptive transfer	Pilot studies in advanced solid tumors (incl. melanoma)	Demonstrated feasibility; limited durable responses	Nicol et al., 2011 (PMCID: PMC3171009)
2000s	Zoledronate + IL‐2 systemic infusion	Aminobisphosphonates induce IPP accumulation → Vγ9δ2 activation	Phase I/II trials in advanced melanoma & RCC	Robust γδ expansion; safe; but clinical responses rare	Kunzmann et al., 2012 (PMID: 22246681)
2010	BrHPP/IPH1101 (synthetic phosphoantigen) + low‐dose IL‐2	Pharmacologic γδ T‐cell activation via BTN3A/IPP axis	Phase I; 28 pts. with advanced solid tumors (incl. melanoma)	Well tolerated; potent Vγ9δ2 expansion; limited tumor shrinkage	NCT00582790; Clin Cancer Res 2010 (PMID: 20563721)
2010–2011	Adoptive transfer of Vγ9δ2 T cells + zoledronate infusion	Ex vivo expanded γδ cells + systemic phosphoantigen boost	Phase I (18 pts.; incl. melanoma, UK)	Safe; γδ cells trafficked to tumors; most progressed; few responses when combined with prior therapy	NCT01017705; Br J Cancer 2011 (PMCID: PMC3171009)
2012	Zoledronic acid + low‐dose IL‐2	In vivo γδ2 activation	Prospective I/II (21 pts.; RCC, melanoma, AML)	Strong γδ activation; no RECIST responses in melanoma/RCC; 2 PRs in AML; VEGF correlated with resistance	J Immunother 2012 (PMID: 22130169)
2015	IL‐15 cytokine infusion	Systemic IL‐15 expands NK and γδ compartments	Phase I, metastatic melanoma & solid tumors	Potent γδ/NK expansion; no objective monotherapy responses	Conlon et al., 2015; NCT01021059
2019–2023 (preclinical)	γδ‐redirected CAR‐T & bispecific engagers	γδ engineered with CAR or bsTCE against melanoma antigens	Xenograft melanoma models	Superior cytotoxicity vs. αβ CARs; prevented tumor escape	Silva‐Santos et al., 2019; Sebestyen et al., 2020
2020–2023	BTN3A agonist ICT01 (± pembrolizumab; EVICTION study)	Pharmacologic Vγ9δ2 activation via BTN3A → tumor trafficking	CPI‐refractory advanced melanoma	Objective responses incl. CR in brain mets; tolerable	NCT04243499 (ASCO/SITC data)
2022–2023	ICT01 + low‐dose IL‐2 (EVICTION‐2)	BTN3A agonism + cytokine expansion	Advanced solid tumors	No DLTs; reproducible γδ + NK expansion; efficacy readouts pending	NCT05307874
2023	EGFR‐targeted γδ engager PF‐08046052/LAVA‐1223	Redirects Vγ9δ2 to EGFR+ tumors	Advanced EGFR+ solid tumors (basket incl. SCC, melanoma subsets)	Ongoing; early dose escalation	NCT05983133
2024	Lifileucel (Iovance TIL therapy)	Polyclonal TILs with incidental γδ infiltration	FDA‐approved in post‐ICI melanoma	Validates adoptive cell therapy as standard; highlights γδ role within TILs	Sarnaik et al., 2021 (PMID: 33502949)
2024–2025	ACE2016 (allogeneic γδ2 cells + cetuximab; ACC platform)	Engineered γδ cells targeting EGFR	Early‐phase basket trial incl. cutaneous SCC; melanoma cohorts possible	IND cleared 2024; escalation ongoing	NCT06415487
2025	CAR‐γδ T cell basket trials (Claudin18.2, CD123, CD20)	Genetically engineered γδ recognizing tumor antigens	Hematologic + solid tumors (melanoma preclinical data strong)	Preclinical cytotoxicity promising; first‐in‐human trials ongoing	Pablo et al., 2023 (review)

Over the past decade, ICIs have transformed the management of advanced melanoma. Anti–PD‐1 antibodies such as pembrolizumab and nivolumab have significantly improved overall survival, and pembrolizumab exhibited lower toxicity rates than did ipilimumab for treatment of advanced melanoma [59]. Despite these advances, approximately half of patients either fail to respond initially or eventually relapse. Mechanisms of primary, adaptive immune, and acquired resistance include loss of antigen presentation, defects in IFN‐γ signaling, and profound immunosuppression within the TME, which also drives T‐cell exhaustion [60]. Furthermore, immune‐related adverse events (irAEs), such as colitis and hepatitis, remain a significant limitation of ICI therapy [61]. These challenges highlight the need for novel strategies that can enhance response rates, overcome resistance, and reduce toxicity.

#### 3.10.1 Clinical Applications: Combinations With ICIs

The combination of γδ T‐cell–based therapies with ICIs represents a rational strategy to improve antitumor efficacy while maintaining safety. Several mechanisms support this approach. First, activation of γδ T cells,e.g., following stimulation with aminobisphosphonates or phosphoantigens, leads to up‐regulation of PD‐1, creating a biological need for concurrent PD‐1 blockade. In preclinical studies, PD‐1 inhibition restored γδ T‐cell proliferation, IFN‐γ production, and cytotoxicity within the tumor microenvironment [62]. Second, PD‐1 expression on tissue‐adapted Vδ1+ γδ T cells defines a distinct functional state rather than terminal exhaustion. In both melanoma and NSCLC samples, PD‐1+ Vδ1+ cells exhibited suppressed activity that could be undone by PD‐1/PD‐L1 blockade such as atezolizumab, even when tumor antigen levels were low, highlighting their potential for adoptive transfer strategies [63]. Finally, γδ T cells contribute unique mechanisms not shared by αβ T cells: They mediate MHC‐independent cytotoxicity, secrete IFN‐γ to “heat up” the tumor microenvironment, and cross‐present tumor antigens to conventional T cells. Together, these functions complement ICIs by broadening immune responses and may start to address resistance mechanisms [64].

Early pharmacologic strategies to activate γδ T cells—including aminobisphosphonates or phosphoantigens, with or without IL‐2—consistently expanded circulating Vγ9δ2 populations but produced limited clinical benefit in melanoma when given as monotherapy. These outcomes reinforce the rationale for combining γδ T‐cell activation with ICIs, cytokines, or targeted agents [65].

Clinical evidence suggests that therapeutic benefit may be subset dependent. In particular, Vδ1+ γδ T cells—tissue‐adapted populations enriched in tumors—have been associated with favorable responses to PD‐1 blockade. Transcriptomic detection of TRDV1, encoding the Vδ1 TCR δ chain, predicted benefit from anti–PD‐1 therapy in melanoma, particularly in tumors with low neoantigen burden. These findings highlight γδ T‐cell heterogeneity as both a determinant of therapeutic efficacy and a potential predictive biomarker in ICI therapy [63].

Multiple preclinical studies and early‐phase clinical trials are shown in Table 2. A study by Zaretsky et al. demonstrated that resistance to PD‐1 blockade therapy in melanoma patients has been found to be correlated with interferon signaling and antigen presentation [66]. Previous studies have also shown that HLA class I downregulation or loss of β2‐microglobulin limits melanoma ICI blockade. γδ‐redirecting platforms such as EGFR‐targeted bispecific engagers (e.g., LAVA‐1223, ACE2016) are entering clinical testing. Cutaneous squamous cell carcinoma has been prioritized due to its high EGFR expression, with early data supporting safety and γδ T cell engagement [67]. These approaches highlight the feasibility of combining γδ activation with PD‐1 blockade to improve responses in solid tumors, including melanoma.

### 3.11 Cellular Therapies Based on γδ T Cells in Cutaneous Malignancies

The adoptive transfer of ex vivo induced γδ T cell subtypes (more commonly Vγ9δ2 and Vδ1 subtypes) in addition to the engineering of γδ T cells to express specific cell surface receptors are techniques that have been utilized to treat solid tumors and hematologic malignancies. Li et al. engineered γδ T cells to express a CD19‐specific T cell receptor for the treatment of patients with refractory or relapsed diffuse large B cell lymphoma (DLBCL). This treatment achieved a clinical response among the majority of patients (with complete remission among a subset) with a median follow‐up period of almost 3 years, thus highlighting the potential utility of engineering γδ T cells to target specific malignant populations [68].

When engineered with chimeric antigen receptors (CARs), γδ T cells may be a promising therapy for melanoma. Compared with conventional αβ T cells, γδ T cells have distinct biological advantages. Many solid tumors—including melanoma—evade αβ T‐cell surveillance by downregulating MHC molecules or altering antigen presentation and thus limit the efficacy of αβ CAR‐T cells [69]. In contrast, γδ T cells recognize stress‐induced ligands, phosphoantigens, and non‐peptide tumor‐associated antigens in an MHC‐independent fashion, allowing them to bypass melanoma's immune‐escape strategies [56]. Unlike αβ TCRs, which recognize polymorphic MHC molecules and can trigger alloreactivity, γδ T cells form a smaller, unconventional subset that bridges innate and adaptive immunity. Their MHC independence feature not only minimizes GVHD risk but also supports “off‐the‐shelf” allogeneic CAR‐T applications with fewer toxicities [65]. In addition, γδ T cells naturally infiltrate epithelial and mucosal tissues where melanoma and other solid tumors arise. This tissue tropism enhances tumor infiltration, a major barrier in solid‐tumor CAR‐T therapy, and makes γδ T cells particularly suited for epithelial‐origin cancers [70]. Moreover, γδ T cells exert direct cytotoxicity via pathways such as Fas/FasL and perforin while also engaging broader immune responses through crosstalk with NK cells, dendritic cells, and αβ T cells, thereby amplifying antitumor immunity [71]. Indeed, aggregation of targeted FasL has previously been demonstrated to be an effective cytotoxic agent against cancer cells [72].

The relevance of γδ T cells is also evident in the evolving landscape of melanoma adoptive cell therapies. Lifileucel, the first FDA‐approved TIL therapy for advanced melanoma in 2024, highlights the potential of polyclonal T‐cell products [24, 73]. While dominated by CD8+ αβ T cells, TIL products often contain γδ T cells, which can contribute MHC‐independent cytotoxicity and cross‐priming of αβ responses [56]. Clinical‐grade analyses demonstrated that Vδ1 subsets expand under GMP conditions, exhibit MHC‐unrestricted tumor recognition, and can be safely co‐infused with αβ TILs, supporting the feasibility of incorporating γδ subsets into modern TIL protocols [74, 75]. These findings suggest γδ T cells not only augment TIL efficacy but may also serve as biomarkers of durable responses [26].

In summary, γδ T‐cell–based strategies represent a promising but still early‐stage approach in melanoma. Their unique biology reduced risk of GVHD and capacity to function independently of MHC make them strong candidates for both engineered CAR applications and next‐generation TIL products. Future development will likely depend on subset‐specific engineering, streamlined manufacturing, and rational combination regimens—such as pairing γδ CARs with checkpoint inhibitors or neoantigen vaccines—to maximize therapeutic durability and broaden their clinical applicability.

## 4 Discussion

γδ T cells traditionally recognize antigens independent of MHC presentation. Recent work by Benveniste et al. reported the ability to generate γδ T cells in vitro, capable of recognizing antigen presented by MHC. These cells were derived from hematopoietic progenitor cells or cloned from peripheral naïve T cells, thus offering great potential as a future immunotherapeutic [64]. Additionally, γδ T cells may offer prognostic significance in these cutaneous neoplasms through their quantification as well as functional state.

Our current understanding of immune regulation has allowed melanoma therapy to progress significantly with the introduction of immune checkpoint inhibitors, yet resistance and relapse remain major challenges. Γδ T‐cell–based strategies are emerging as an investigational therapeutic option. Early studies, while still limited, demonstrate encouraging activity in hematological malignancy and solid tumors including melanoma. Though antitumor function predominantly stems from MHA class I‐restricted CD8+ T‐cell responses, the advantages of γδ T cells include their ability to recognize and eliminate tumor cells in an MHC‐independent manner, produce proinflammatory cytokines that enhance tumor immunogenicity, and cross‐prime adaptive responses. Increasing numbers of preclinical and early‐phase clinical trials are now evaluating combinatorial approaches of ICIs and γδ T cell therapies. However, challenges remain, including the possibility of protumor IL‐17–producing subsets and the need for strategies to overcome tumor‐mediated suppression. Additionally, we acknowledge the modest clinical activity of this subset of cells in prior studies and that γδ phenotypic subsets have been shown to be context‐dependent. Future studies should focus on characterizing γδ T cell subsets, refining combinatorial strategies with ICIs, further characterizing and addressing the immunosuppressive effects of the TME, and defining biomarkers to identify patients most likely to benefit. Their unique characteristics and functional properties may help influence future immunotherapeutics.

## Conflicts of Interest

Dr. Du reports research support from the Patient Care Trust Fund (PCTF) at NYC Health + Hospitals/Jacobi. All other authors declare no conflicts of interest.

## Data Availability

Data sharing not applicable to this article as no datasets were generated or analysed during the current study.
